# Comparison of the Vaginal Microbiomes of Premenopausal and Postmenopausal Women

**DOI:** 10.3389/fmicb.2019.00193

**Published:** 2019-02-14

**Authors:** Karol Gliniewicz, G. Maria Schneider, Benjamin J. Ridenhour, Christopher J. Williams, Yuli Song, Miranda A. Farage, Kenneth Miller, Larry J. Forney

**Affiliations:** ^1^Department of Biological Sciences, University of Idaho, Moscow, ID, United States; ^2^Institute for Bioinformatics and Evolutionary Studies, University of Idaho, Moscow, ID, United States; ^3^Department of Statistical Science, University of Idaho, Moscow, ID, United States; ^4^The Procter and Gamble Company, Cincinnati, OH, United States

**Keywords:** menopause, vaginal microbiome, vaginal atrophy, hormone replacement therapy, microbial community structure and function, premenopause

## Abstract

For decades hormone therapy (HT) has been prescribed to treat the symptoms of menopause, such as vaginal dryness, itching and burning. Here we sought to compare the vaginal microbiomes of postmenopausal women who received low dose estrogen therapy to those of premenopausal and postmenopausal women, and to do so in conjunction with assessing the alleviation of symptoms associated with vaginal atrophy. In this study vaginal swab samples were obtained from 45 women who were classified as either premenopausal, postmenopausal, or postmenopausal and undergoing HT. The vaginal microbiomes of these women were characterized by *16S rRNA* gene sequencing and bacterial abundances were quantified by qPCR. We found that the vaginal communities from our cohort could be divided into six clusters (A-F) based on differences in the composition and relative abundances of bacterial taxa. Communities in cluster A were dominated by *Lactobacillus crispatus*, and those of cluster B were dominated by *Gardnerella vaginalis*. Communities in cluster C had high proportions of *L. iners*, while those in cluster D were more even and included several co-dominant taxa. Communities in clusters E and F were dominated by *Bifidobacterium* and *L. gasseri*, respectively. The vaginal communities of most postmenopausal women receiving HT (10/15) were dominated by species of lactobacilli and belonged to clusters A, C, and F (*P* < 0.001). This sharply contrasts with vaginal communities of postmenopausal women without HT, most of which (10/15) were in cluster D, depleted of lactobacilli, and had about 10-fold fewer total bacteria (*P* < 0.05). The vaginal communities of women in each study group differed in terms of the dominant bacterial species composition and relative abundance. Those of postmenopausal women receiving HT significantly differed from those of postmenopausal women without HT and were most often dominated by species of *Lactobacillus*. Noteworthy, HT greatly improved vaginal atrophy scores, decreased vaginal pH, and significantly increased bacterial numbers in comparison to postmenopausal women not receiving HT.

## Introduction

Menopause usually occurs in the fourth or fifth decade of life and is defined as cessation of menstruation for 12 consecutive months marking the end of fertility. Decreased ovary function during menopause results in lower levels of circulating estrogen, which often leads to various symptoms such as hot flashes, night sweats, decreased cognitive functions and mood changes that are experienced by women during the years immediately preceding and during menopause (Takahashi and Johnson, 2015). Additionally, genitourinary tract changes associated with vulvovaginal atrophy (VVA) are common and affect at least 50% of menopausal women (Santoro and Komi, 2009; Nappi and Kokot-Kierepa, 2010, 2012; Simon et al., 2013; Minkin et al., 2014). The most common signs of VVA include dryness, redness, itching, and dyspareunia, with occasional discharge and/or bleeding (Bachmann and Nevadunsky, 2000). For decades, both systemic and local low-dose estrogen therapy have been widely used for management of VVA and vaginal dryness in postmenopausal women (Raymundo et al., 2004; Bachmann et al., 2008; Pinkerton et al., 2009).

Changes in the vaginal environment during menopause are accompanied by changes in the species composition of the vaginal microbiome (Hillier and Lau, 1997; Brotman et al., 2014; Shen et al., 2016). Often this is manifest in decreased proportions of lactobacilli and lactic acid production, causing an increased vaginal pH that possibly renders the vagina more susceptible to infections and exacerbates the vaginal symptoms associated with VVA. Very few studies have been done to compare the vaginal microbiomes of postmenopausal women who receive HT and those that do not (Yoshimura and Okamura, 2001; Heinemann and Reid, 2005; Shen et al., 2016; Mitchell et al., 2017). Findings show that the communities of women who receive HT often resemble those of premenopausal women by essentially restoring high proportions of *Lactobacillus*, but the means by which this occurs is not understood (Mitchell et al., 2017). In this cross-sectional pilot study, we explicitly compared the vaginal microbiomes of premenopausal women to those of postmenopausal women who receive various forms of HT and those that do not. In addition, we determined bacterial abundance, estimated the species richness and evenness of vaginal communities, and measured various biomarkers of inflammation in the vulvo-vaginal area.

## Materials and Methods

### Subject Data and Sample Collection

Healthy adult women enrolled in the study comprised three groups of 15 females each (Supplementary Table S1 contains detailed demographic data) that were prescreened based on their vaginal pH and vaginal atrophy score as previously described by Farage and coworkers (Farage et al., 2015). Subject recruitment and sample collection were conducted at an independent facility (Radiant Research, Cincinnati, OH). Testing was conducted in compliance with the Good Clinical Practice Regulations [21 Code of Federal Regulations (CFR 50)] and in accordance with the Declaration of Helsinki (WMA Declaration of Helsinki, 2013). Test protocols were approved by the clinical facility’s Institutional Review Board. Subjects were healthy adult female volunteers, 23–67 years of age, who had signed an informed consent. Subjects were excluded from participation if they had had a partial or full hysterectomy, irregular menstrual cycle, skin abnormalities in the vulvar area, diabetes, kidney, heart or circulatory disease, were currently pregnant or breast-feeding, took certain immunosuppressive or anti-inflammatory medications that might interfere with test results, or if they were participating in another clinical study.

To obtain vaginal samples subjects were placed in the dorsolithotomy position, and medically trained personnel used COPAN ESwab^TM^ (COPAN Diagnostics Inc.) to obtain samples from the vaginal wall approximately 2 inches into the vagina. Afterward, the swab was placed in a cryogenic tube and stored in a -80°C freezer until they were shipped on dry ice to the laboratory for further analysis.

### Microbial Community Analysis

We transferred 250 μL of each sample to bead beating tubes and added 100 μL of lytic enzyme cocktail (50 μL lysozyme 500 kU/mL, 6 μL mutanolysin 25 kU/mL, 4 μL lysostaphin 3000 kU/mL, and 41 μL mixture of 10 mM Tris–HCl and 50 mM EDTA pH 8.0). These mixtures were incubated at 37 °C for 1 h in a dry heat block. Next, 750 mg of zirconia–silica beads (0.1 mm diameter) were added to all samples, and the tubes were placed in Mini-BeadBeater-96 at room temperature for 1 min at 2100 rpm. Once completed, bead beating was followed by a brief centrifugation. Isolation of bacterial genomic DNA was performed with a QIAamp DNA Mini kit (Qiagen Inc., Valencia, CA, United States) according to the manufacturer’s protocol. DNA concentrations were quantified with a QuantiFluor dsDNA kit (Promega Inc., Madison, WI, United States) using a Turner TBS-380 mini-fluorometer (Turner BioSystems, United States). DNA size and integrity were verified using an Agilent DNA 1000 kit and an Agilent Bioanalyzer 2100 (Agilent, Santa Barbara, CA, United States) using the manufacturer’s protocol.

The V1–V3 region of bacterial *16S rRNA* genes (*Escherichia coli* positions 27F-534R) was amplified using a mixture of degenerate primers that flanked the variable regions (Supplementary Table S2). Amplicons were produced by two consecutive rounds of PCR. The first round of PCR amplified the targeted V1–V3 region of the *16S rRNA* gene, while the second round of PCR attached the sample barcode and sequencing adapters. The concentrations of amplicons were determined using a Pico Green assay (Promega Inc.) and a SpectraMax Gemini XPS fluorometer (Molecular Devices, Sunnyvale, CA, United States), then equal amounts (∼100 ng) were pooled in a single tube. The amplicon pool was cleaned to remove short undesirable fragments using the following procedure. First the pool was size selected with using AMPure beads (Beckman Coulter Inc., Pasadena, CA, United States), the product was then run on a 1% gel, excised from the gel, column purified using a Qiagen MinElute PCR purification kit and size selected again with AMPure beads (Beckman Coulter, Indianapolis, IN, United States). To determine the quality of the amplicons, the pool was PCR amplified with Illumina adaptor specific primers followed by size selection using a DNA1000 chip and an Agilent 2100 Bioanalyzer. The cleaned amplicon pool was then quantified using the KAPA Illumina library quantification kit (KAPA Biosciences) and the Applied Biosystems StepOne plus real-time PCR system. Finally, sequences were obtained using an Illumina MiSeq paired-end 300 bp protocol (Illumina, Inc., San Diego, CA, United States).

Raw DNA sequence reads from the Illumina MiSeq were assigned to samples, demultiplexed and classified in the following manner. The custom python application dbcAmplicons^1^ was used to assign reads to samples based on both expected barcode and primer sequences. Sequencing was performed in the IBEST Genomic Resources Core at the University of Idaho. Barcodes could have at most 1 mismatch (Hamming distance) and primers could have at most 4 mismatches (Levenshtein distance) provided the final 4 bases of the primer matched the target sequence perfectly. Sequence reads were then trimmed of their primer sequence and merged into a single amplicon sequence using the application FLASH (Magoč and Salzberg, 2011). Finally, the RDP Bayesian classifier was used to assign sequences to phylotypes (Wang et al., 2007). Reads were assigned to the first RDP taxonomic level with a bootstrap score ≥50. Reads categorized as belonging to the genus *Lactobacillus* were subsequently analyzed to identify which species of this genus were present in the samples. Blastn was used in BLAST+ (Camacho et al., 2009) to compare each read to a database composed of *16S rRNA* gene sequences longer than 1000 bp belonging to the genus *Lactobacillus* (downloaded from NCBI in February 2014). The identity of the different bacterial species reported as the best match for each read was recorded, the number of reads assigned to each of the taxa counted, and their relative abundance in the different samples calculated. These data were cleaned to include only samples with ≥3,000 reads and those taxa that occurred with a relative abundance of at least 0.01 (1%). All less abundant taxa (below 0.01) were aggregated into the category ‘Other.’ Cleaned data included the relative abundances of 51 OTUs for all 45 test subjects. A species was considered dominant in a given sample or sample cluster, if it reached at least 51% relative abundance.

### Quantifying *16S rRNA* Gene Copy Number

We used a broad-coverage *16S qPCR* assay developed by Liu and coworkers (Liu et al., 2012) to quantify the bacteria in samples. DNA yields from samples were quantified fluorometrically and 0.2 ng of template DNA was added to each 10 μl reaction containing 1.8 μM forward and reverse primer, 225 nM TaqMan^®^ probe, 1× Platinum Quantitative PCR SuperMix-UDG w/ROX (Invitrogen^TM^) and molecular grade water. Each assay included a series of standards and a negative control (no DNA template). All assays were done in triplicate. Cycling parameters were: 3 min at 50°C for treatment with uracil-N glycosylase (UNG), 10 min at 95°C for *Taq* activation, 15 s at 95°C (denaturation), 1 min 60°C (annealing) and extension for 40 cycles. PCR amplification and real-time detection of fluorescence were performed using ABI 7900HT Real Time PCR system with StepOne Plus software (Applied Biosystems^TM^).

### Statistical Analysis and Data Visualization

We visualized the vaginal microbiomes of the subjects using a clustering analysis and a principle coordinates analysis. Both analyses required calculation of dissimilarity between individual samples. Dissimilarity was calculated using the mean absolute difference between samples, $\frac{1}{n}\sum i\left|x_{i}-y_{i}\right|$ where *n* is the number non-zero OTUs in the samples *x* and *y* and *x_i_* and *y_i_* are the relative abundances of the *i*-th OTU in those samples (Anderson et al., 2006). Principle coordinates analysis (Gower, 1966) was performed using a correction factor (Cailliez, 1983; Legendre and Legendre, 1998). To create a dendrogram, complete-linkage clustering was used to perform hierarchical agglomeration of samples (Legendre and Legendre, 1998). The number of clusters within the data was determined by maximizing the silhouette width (a measure of similarity within clusters) with respect to the number of clusters (Rousseeuw, 1987).

Using the clusters detected, we examined the relationship between cluster and treatment as well as cluster and measured traits. A contingency Chi-squared was calculated to test whether or not study groups were evenly distributed among the six clusters. To test for difference among clusters for measured traits (e.g., pH, histamine concentrations), we used one-way ANOVA with log-transformed dependent variables and the assigned cluster as the explanatory variable (vaginal atrophy scores were not log-transformed). Log-transformation was used to ensure the assumption of i.i.d. errors was met in our statistical models. *Post hoc* linear contrasts to compare trait means of clusters using Tukey’s range test (Tukey, 1949).

Similar to the analyses of trait means for clusters, one-way ANOVA with treatment as the explanatory factor and *post hoc* linear contrasts were used to compare DNA concentrations extracted from samples and for estimation of *16S rRNA* gene copies with BactQuant qPCR TaqMan assay (Liu et al., 2012). *Post hoc* linear contrasts were corrected for multiple comparisons using the method proposed by Holm (1979).

## Results

Of the women enrolled in the study (Supplementary Table S1), one group included only premenopausal women (PRE), mean age of 33 (±6.4) years, with a vaginal pH ≤ 5 and vaginal atrophy score ≤2. The second group included only postmenopausal women (POST) with a vaginal pH ≥ 5 and vaginal atrophy score ≥6, while the third group included postmenopausal women undergoing hormone therapy (POST+HT) who had a vaginal pH ≤ 5 and a vaginal atrophy score ≤2. Women in the POST+HT group had received HT in some form (oral, vaginal, or transdermal patch) for at least 12 consecutive months prior to the study. The mean age of postmenopausal women enrolled in the study was 60.5 years, and height, weight and BMI were comparable among the groups.

### Vaginal Bacterial Community Composition

**FIGURE 1:**
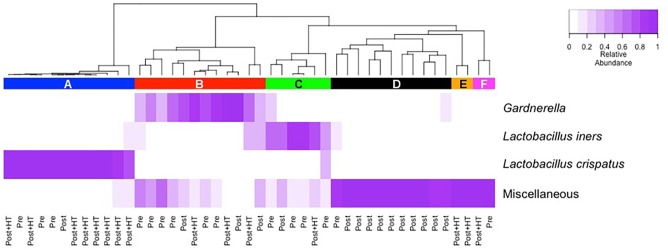
Cluster analysis of vaginal bacterial communities found in 45 women who were either premenopausal, postmenopausal, or postmenopausal and receiving hormone replacement therapy. The dendrogram was created as described in the Materials and Methods section. The number of clusters within the data was determined by maximizing the silhouette width as described by Rousseeuw (1987).

**FIGURE 2:**
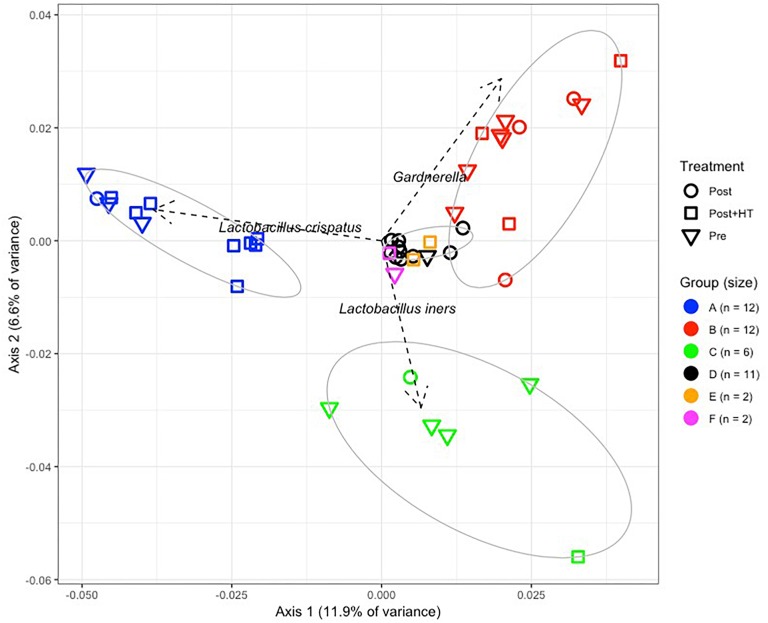
Principle components analysis of vaginal bacterial communities found in 45 women who were either premenopausal, postmenopausal, or postmenopausal and receiving hormone replacement therapy.

We used culture-independent methods to characterize vaginal bacterial communities in these three groups of women. This was done by classifying sequences of the V1–V3 region of bacterial *16S rRNA* genes in vaginal swab samples. Hierarchical clustering of bacterial community composition data showed that the 45 vaginal samples could be assigned to six distinct clusters based on differences in the composition of relative abundances of bacterial taxa (Figure 1 and Supplementary Tables S3–S5). Clustering was dependent on study group (*χ*^2^ = 32.55, *df* = 10, *P*-value < 0.001). With the exception of cluster D, the rank-abundance curves of bacterial taxa were highly skewed and communities within clusters were dominated by a single species. The average proportion of *L. crispatus* in cluster A (*N* = 12) was 0.92 (95% CI: 0.92, 0.92), while the average proportion of *Gardnerella* in cluster B (*N* = 12) was 0.66 (95% CI: 0.65, 0.66), and that of *L. iners* in cluster C (*N* = 6) was 0.73 (95% CI: 0.72, 0.73). The communities in clusters E and F were dominated by *Bifidobacterium* and *L. gasseri*, respectively; each with *N* = 2. Finally, communities in cluster D (*N* = 11) exhibited greater diversity and evenness and included several co-dominant taxa, including *Anaerococcus*, *Atopobium*, *Finegoldia*, *Gardnerella*, *Prevotella*, and *Streptococcus.* The compositions of all samples were visualized by principle coordinates analysis (Figure 2), which illustrates the clear distinction between communities that were dominated by either *L. crispatus*, *L. iners*, or *G. vaginalis*.

**FIGURE 3:**
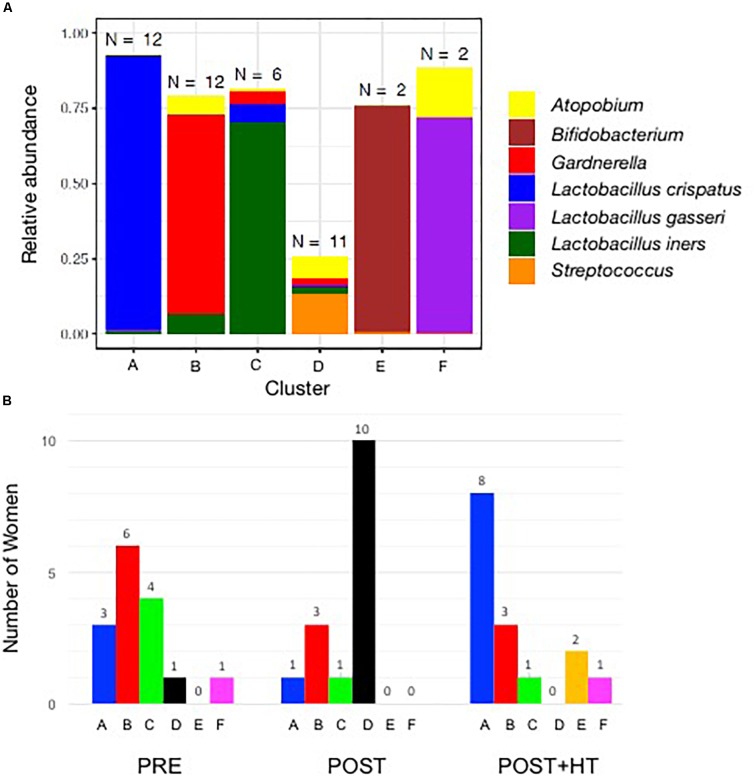
**(A)** Stacked bar chart showing the predominant taxa that comprised >10% (on average) of communities in at least one cluster. The number of women in each cluster is shown above each bar, and the identity of the taxa are shown in the key on the right of the graph. **(B)** Number of women in clusters A–F in the three treatment groups. One group (PRE) included only premenopausal women with a vaginal pH ≤ 5 and vaginal atrophy score ≤2. The second group (POST) included only postmenopausal women with a vaginal pH ≥ 5 and vaginal atrophy score ≥6, while the third group (POST+H) included postmenopausal women undergoing hormone replacement therapy who had a vaginal pH ≤ 5 and a vaginal atrophy score ≤2.

The vaginal communities of most postmenopausal women undergoing HT were dominated by lactobacilli, namely *L. crispatus* (8/15), *L. iners* (1/15), and *L. gasseri* (1/15) and belonged to clusters A, C, and F, respectively (Figure 3A,B). The remaining five communities of women receiving HT had high proportions of either *Gardnerella* (3/15; cluster B) or *Bifidobacterium* (2/15; cluster E). In contrast, the vaginal communities of postmenopausal women not receiving HT were very different and few were dominated by species of *Lactobacillus* (2/15). Instead, they very often included various strictly anaerobic bacteria (cluster D; 10/15) and were sometimes dominated by *Gardnerella* (cluster B; 3/15). Of the 15 premenopausal women in this study only eight had vaginal communities dominated by species of *Lactobacillus*, with three dominated by *L. crispatus*, four dominated by *L. iners* and one dominated by *L. gasseri.*

### Quantification of Bacterial Number in Vaginal Communities

**FIGURE 4:**
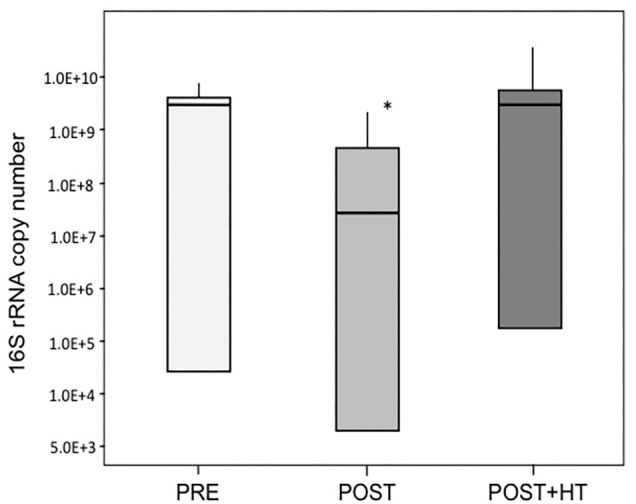
Average *16S rRNA* gene copy number in samples of women in the three treatment groups. One group (PRE) included only premenopausal women with a vaginal pH ≤ 5 and vaginal atrophy score ≤2. The second group (POST) included only postmenopausal women with a vaginal pH ≥ 5 and vaginal atrophy score ≥6, while the third group (POST+H) included postmenopausal women undergoing hormone replacement therapy who had a vaginal pH ≤ 5 and a vaginal atrophy score ≤2. Asterisk denotes statistically significant difference (*P* < 0.05), medians are indicated by thicker lines within the bar graph for each group of women.

We used qPCR to quantify the number of *16S rRNA* gene copies in vaginal samples and these data were used as a proxy for estimating the abundance of bacteria present in vaginal samples (Figure 4). The average number of *16S rRNA* genes, and therefore the number of bacteria, varied widely among women in all three study groups. However, on average the number of bacteria in samples from postmenopausal women receiving HT was nearly the same as in samples from premenopausal women. In contrast, the abundance of bacteria in postmenopausal women was roughly 10-fold lower than the other groups (ANOVA; *F*-ratio = 4.18, *P* < 0.05). These differences in bacterial abundance were consistent with the observation that, on average, significantly less genomic DNA was obtained from samples from postmenopausal women not receiving HT (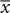 = 1.99 ± 0.61 ng/μl) as compared to samples from postmenopausal women who did receive HT (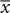 = 9.25 ± 1.63 ng/μl) or premenopausal women (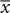 = 5.59 ± 1.35 ng/μl) (ANOVA, *F*-ratio = 8.13, *P* < 0.005).

### Effect of Estrogen Replacement Therapy on Vaginal Atrophy and Biochemical Markers

Farage et al. (2015) used clinical data from these same study subjects to characterize anatomical and physiological changes to the vulvovaginal region that are associated with menopause. Their analyses focused on differences among study groups, whereas here we explored differences among women based on the vaginal microbiome. One-way ANOVA of vaginal atrophy scores and quantified biochemical markers versus cluster membership revealed significant differences among clusters in 8 of the 23 measurements (Table 1). Pairwise comparison of cluster means using Tukey’s HSD revealed a general pattern for the eight measurements where significant differences were found. In general, women belonging to cluster D had lower histamine levels, a greater ratio of histidine to histamine, a lower IL-1rα to IL-1α ratio, higher pH and higher vaginal atrophy scores (Supplementary Tables S6, S7).

**Table 1:** Pairwise comparison of clusters based on different physiological and biochemical factors.

Variable	*p*	Pairwise differences (*p*)
Histamine (labia majora)	0.04	B > D (0.09)
Histamine (labia minora)	0.04	B > D (0.03)
Histidine:Histamine (labia majora)	0.05	
Histidine:Histamine (labia minora)	0.08	D > B (0.09)
IL-1rα:IL-1α (labia minora)	0.01	A > D (0.01); B > D (0.01); D > C (0.005)
Vaginal atrophy	<0.001	D > A (<0.001); D > B (<0.001); D > C (0.005)
Vaginal pH (mid-vagina)	<0.001	B > A (0.02); B > C (0.1); D > A (<0.001); D > B (<0.001); D > C (<0.001)
Vaginal pH (labia minora)	0.02	D > A (0.01)


*Model results were not significant for histamine (introitus), histamine:histidine (introitus), histidine (introitus, labia majora, labia minora), IL-1rα:IL-1α (introitus and labia majora), natural moisturizing factor (introitus, labia majora, and labia minora), temperature (introitus, labia majora, and labia minora), and pH (introitus and labia majora). Clusters: A, dominated by Lactobacillus crispatus; B, Gardnerella vaginalis; C, L. iners; D, several co-dominant taxa.*

## Discussion

In this study, we characterized the vaginal bacterial communities of women in three groups: postmenopausal women undergoing hormone replacement therapy who had a vaginal pH ≤ 5 and a vaginal atrophy score ≤2; postmenopausal women with a vaginal pH ≥ 5 and vaginal atrophy score ≥6, and premenopausal women with a vaginal pH ≤ 5 and vaginal atrophy score ≤2. The vaginal communities of women in each group markedly differed from one another in terms of bacterial species composition. Among all women there were six sorts of communities that could be distinguished based on the kinds and relative abundance of the dominant bacterial species. Four of these accounted for most of the community types found in the women studied, and each could be characterized by the bacterial taxa that were dominant. No single community type was exclusively found in a single study group. Indeed, it was quite the opposite. For example, in premenopausal women three sorts of communities were commonly found (13/15) that were dominated by either *L. crispatus*, *G. vaginalis*, or *L. iners* and clustered in clusters A, B, and C, respectively. These same community types also occurred in postmenopausal women and postmenopausal women undergoing HT, although their frequency varied. Despite the relatively small study cohort these data suggest that the species composition of vaginal communities cannot be used to predict membership in a treatment group.

The vaginal communities of most postmenopausal women receiving HT were dominated by species of lactobacilli (10/15), whereas this was usually not the case in untreated postmenopausal women (3/15). The preponderance of lactobacilli in the vaginal communities of women receiving HT has been previously reported in several studies and is correlated with the alleviation of symptoms associated with VVA (Ginkel et al., 1993; Devillard et al., 2004; Mac Bride et al., 2010; Lethaby et al., 2016; Shen et al., 2016; Mitchell et al., 2017). Our findings suggest that HT may lead to preferential enrichment of *L. crispatus* and not other species of *Lactobacillus.* This could represent an additional benefit of HT therapy since among the vaginal lactobacilli it is thought that *L. crispatus* most effectively reduces the risk to sexually transmitted infections and various adverse gynecological sequelae (Barrons and Tassone, 2008; Verstraelen et al., 2009; Stapleton et al., 2011). The underlying circumstances that lead to the enrichment of *L. crispatus* are completely unknown. It has been suggested that estrogen stimulates that proliferation of squamous epithelial cells, which is accompanied by the increased production of glycogen by these cells (Cruickshank, 1934; Buchanan et al., 1998; Krause et al., 2009; Sturdee et al., 2010). Glucose, maltose and maltodextrins produced through the hydrolysis of glycogen are thought to serve as carbon sources that support the proliferation of vaginal lactobacilli. The simplicity of this model is attractive, but it leaves important questions unanswered. These include the source(s) of amylase(s) needed for the depolymerization of glycogen, how lactobacilli effectively compete for these “common goods” that are available to all community members, and whether all vaginal lactobacilli compete equally well for these resources. The last of these seems unlikely. Our findings hint at the possibility that *L. crispatus* may disproportionally benefit from an increase in the production of glycogen or certain other resources produced by the host in response to HT. However, the underlying drivers of vaginal community composition and the basis for differences among women of all ages is unknown.

This study confirms that vaginal bacterial communities of postmenopausal women are usually not dominated by species of *Lactobacillus* (Heinemann and Reid, 2005; Gustafsson et al., 2011; Mirmonsef et al., 2015). In the present study, the communities of just 3 of 15 women were dominated by lactobacilli, which is good agreement with the findings of Shen et al. (2016) who reported that this was the case in only seven of thirty women (when dominance was defined as >25% of a community). It should be noted that various authors describe the vaginal communities of postmenopausal women as being “depleted” of lactobacilli (Hillier and Lau, 1997; Burton and Reid, 2002; Pabich et al., 2003; Zhang et al., 2012) and imply that these communities are rather consistent in terms of species composition. These rather general statements seem misleading. Instead, our findings indicate that the vaginal communities of postmenopausal women most often include high proportions of strictly anaerobic bacteria that are co-dominant (10/15, cluster D), but vary among women, which is consistent with the findings of others (Milsom et al., 1993; Cauci et al., 2002; Shen et al., 2016). While the composition of these communities resembles those of premenopausal women with bacterial vaginosis diagnosed by Nugent criteria (Ravel et al., 2011, 2013; Jung et al., 2017), all subjects were asymptomatic and free of clinical signs of dysbiosis. In general, women belonging to this group had lower histamine levels, a greater ratio of histidine to histamine, a lower IL-1rα to IL-1α ratio, higher pH and higher vaginal atrophy scores. Presently we cannot explain why communities with this composition are sometimes associated with vaginal symptoms such as itching or discharge, while in other cases they are not. However, two possibilities would be genetic differences within bacterial species wherein some strains elicit symptoms while others do not, underlying genetic or physiological differences in subpopulations of women, or a combination of these two. Additionally, the cytokine, histidine/histamine and inflammatory marker measurements have been collected using vulvar skin, which may reflect some general menopause symptoms, rather than directly relate to the vaginal environment. This aspect is one of the limitations of this study. Other limitations would include relatively small sample size and racial diversity or measured estrogen levels, which might have contributed to not being able to detect some of the significant differences regarding the correlations between biomarkers and vaginal microbiota.

Our findings resemble those of previous reports showing the relatively low proportions of lactobacilli in the vaginal communities of postmenopausal women (Hummelen et al., 2011; Brotman et al., 2014). This might be attributed to low estrogen during menopause that results in atrophy and thinning of the vaginal squamous epithelium and decreased vaginal secretions. We suspect that this also leads to lower levels, and possibly differences in the kinds of nutrients that are available to support the growth of vaginal bacteria. This may well create a less favorable host environment for vaginal microbiota to thrive, especially the vaginal lactobacilli (Mirmonsef et al., 2015). The lower number of bacteria found in samples from postmenopausal women who were not receiving low dose estrogen therapy is consistent with this postulate and the findings of Hillier and Lau (1997). Low-dose local estrogen formulations effectively relieve the symptoms of vulvovaginal atrophy in most, but not all women (Robinson and Cardozo, 2003; Galhardo et al., 2006; Shen et al., 2016; Mitchell et al., 2017). This is most often accompanied by markedly increased proportions of lactobacilli that often exceed 90%, but this is not always the case (Pabich et al., 2003). The reasons for this incongruence are unknown. It could result from genetic or physiological differences between hosts that might be overcome by personalizing the dosage, changing the mode of estrogen administration, the estrogens used, or the timing of treatments (Archer, 2010). Personalizing HT may provide a way to more consistently reshape the vaginal microbiome of women, so they are dominated by lactobacilli while alleviating the symptoms of VVA.

## Author Contributions

KG was involved in analysis of the microbiome data, performing *16S qPCR* assays, and in preparation of the manuscript. GS extracted gDNA from samples, run QC analyses, and prepared them for DNA sequencing. BR and CW were responsible for statistical analyses of the data and contributed to preparation of the manuscript. YS, MF, and KM were involved in sample collection and study design and contributed to the manuscript preparation. LF was involved in manuscript editing and coordination of all parties engaged in the study.

## Conflict of Interest Statement

YS, MF, and KM were employed by Procter and Gamble Company. LF has consulted for Procter and Gamble Company. The remaining authors declare that the research was conducted in the absence of any commercial or financial relationships that could be construed as a potential conflict of interest.

## Footnotes

1 https://github.com/msettles/dbcAmplicons
