# Supplementary material for: Comparison of the Vaginal Microbiomes of Premenopausal and Postmenopausal Women
Source: Front Microbiol. 2019 Feb 14;10:193. doi: 10.3389/fmicb.2019.00193 (PMC6382698; doi:10.3389/fmicb.2019.00193)
Supplement: Supplementary file 1 [file Table_1.PDF]

Table S1. Demopgraphics of women enrolled in study.

Parameter	Premenopausal (N=15)	Postmenopausal (N=15)	Postmenopausal + HT (N=15)
Age (years)	33.0 ± 6.4[a]	60.7 ± 3.6	60.5 ± 3.6
Height (inches)	64.7 ± 3.7	63.5 ± 2.8	63.9 ± 2.5
Weight (lbs)	156 ± 22.8	149.2 ± 27	147.8 ± 23.7
BMI	26.2 ± 3.3	25.9 ± 3.6	25.5 ± 4.3
Years since last period	NA	15.8 ± 9.3	14.2 ± 8.2
Average time on HT	NA	NA	5y, 2 mo
Ethnicity			
African-American	6 (40%)	2 (13%)	0 (0%)
Caucasian	8 (53%)	13 (87%)	15 (100%)
Other	1 (7%)	0 (0%)	0 (0%)

[a] mean ± standard deviation
HT indicates hormone therapy
