# Supplementary material for: Comparison of the Vaginal Microbiomes of Premenopausal and Postmenopausal Women
Source: Front Microbiol. 2019 Feb 14;10:193. doi: 10.3389/fmicb.2019.00193 (PMC6382698; doi:10.3389/fmicb.2019.00193)
Supplement: Supplementary file 2 [file Table_2.PDF]

Table S2. Primers used for amplification of V1-V3 variable regions.

NAME	SEQUENCE
534R_YM1-F	ACACTGACGACATGGTTCTACAGTAGAGTTTGATCCTGGCTCAG
534R_YM2-F	ACACTGACGACATGGTTCTACACGTAGAGTTTGATCATGGCTCAG
534R_YM3-F	ACACTGACGACATGGTTCTACAACGTAGAGTTTGATTCTGGCTCAG
534R_YM4-F	ACACTGACGACATGGTTCTACATACGTAGAGTTTGATTATGGCTCAG
534R_Bif-F	ACACTGACGACATGGTTCTACAGTACGTAGGGTTCGATTCTGGCTCAG
534R_Bor-F	ACACTGACGACATGGTTCTACACGTACGTAGAGTTTGATCCTGGCTTAG
534R_Chl-F	ACACTGACGACATGGTTCTACAACGTACGTAGAATTTGATCTTGGTTCAG
27F_1-R	TACGGTAGCAGAGACTTGGTCTCCATTACCGCGGCTGCTGG
27F_2-R	TACGGTAGCAGAGACTTGGTCTGCCATTACCGCGGCTGCTGG
27F_3-R	TACGGTAGCAGAGACTTGGTCTTGCCATTACCGCGGCTGCTGG
27F_4-R	TACGGTAGCAGAGACTTGGTCTATGCCATTACCGCGGCTGCTGG
27F_5-R	TACGGTAGCAGAGACTTGGTCTCATGCCATTACCGCGGCTGCTGG
27F_6-R	TACGGTAGCAGAGACTTGGTCTTCATGCCATTACCGCGGCTGCTGG
27F_7-R	TACGGTAGCAGAGACTTGGTCTATCATGCCATTACCGCGGCTGCTGG
