# Supplementary material for: Comparison of the Vaginal Microbiomes of Premenopausal and Postmenopausal Women
Source: Front Microbiol. 2019 Feb 14;10:193. doi: 10.3389/fmicb.2019.00193 (PMC6382698; doi:10.3389/fmicb.2019.00193)
Supplement: Supplementary file 3 [file Table_3.pdf]

Table S3. Relative abundance of bacterial taxa based on 16S *rRNA* gene sequencing in vaginal samples obtained from premenopausal women (PRE).

	**Subject**														
**Taxon name**	**1003**	**1005**	**1014**	**1015**	**1017**	**1021**	**1022**	**1025**	**1026**	**1029**	**1030**	**1031**	**1032**	**1033**	**1036**
Atopobium	0.23	0.04	0.00	0.00	0.06	0.00	0.00	0.00	0.09	0.02	0.08	0.00	0.01	0.02	0.00
Coriobacteriaceae	0.03	0.00	0.00	0.00	0.00	0.00	0.00	0.00	0.00	0.00	0.00	0.00	0.00	0.01	0.00
Gardnerella	0.36	0.22	0.00	0.00	0.81	0.01	0.00	0.00	0.79	0.33	0.06	0.02	0.54	0.66	0.00
Lachnospiraceae	0.11	0.00	0.00	0.00	0.03	0.00	0.00	0.00	0.00	0.29	0.22	0.00	0.32	0.14	0.00
Lactobacillaceae	0.00	0.01	0.01	0.00	0.00	0.01	0.00	0.00	0.00	0.00	0.00	0.00	0.00	0.00	0.00
Lactobacillales	0.00	0.01	0.01	0.00	0.00	0.01	0.00	0.00	0.00	0.00	0.00	0.01	0.00	0.00	0.00
Lactobacillus	0.00	0.07	0.09	0.05	0.00	0.09	0.05	0.04	0.00	0.01	0.01	0.08	0.00	0.00	0.04
Lactobacillus crispatus	0.00	0.00	0.00	0.00	0.00	0.00	0.93	0.94	0.00	0.00	0.00	0.36	0.00	0.00	0.94
Lactobacillus gasseri	0.00	0.00	0.00	0.93	0.00	0.00	0.00	0.00	0.00	0.00	0.00	0.00	0.00	0.00	0.00
Lactobacillus iners	0.00	0.63	0.88	0.00	0.02	0.86	0.00	0.00	0.00	0.11	0.13	0.49	0.00	0.00	0.00
Megasphaera	0.03	0.00	0.00	0.00	0.03	0.00	0.00	0.00	0.00	0.05	0.08	0.00	0.04	0.03	0.00
Mobiluncus	0.02	0.00	0.00	0.00	0.00	0.00	0.00	0.00	0.00	0.00	0.02	0.00	0.00	0.00	0.00
Parvimonas	0.05	0.00	0.00	0.00	0.00	0.00	0.00	0.00	0.00	0.01	0.06	0.00	0.00	0.00	0.00
Peptoniphilus	0.00	0.00	0.00	0.00	0.00	0.00	0.00	0.00	0.00	0.00	0.07	0.00	0.00	0.00	0.00
Prevotella	0.04	0.00	0.00	0.00	0.01	0.00	0.00	0.00	0.00	0.04	0.11	0.00	0.00	0.09	0.00
Ruminococcaceae	0.02	0.00	0.00	0.00	0.00	0.00	0.00	0.00	0.00	0.03	0.01	0.00	0.02	0.00	0.00
Saccharofermentans	0.04	0.00	0.00	0.00	0.00	0.00	0.00	0.00	0.00	0.00	0.03	0.00	0.00	0.00	0.00
Sneathia	0.01	0.00	0.00	0.00	0.00	0.00	0.00	0.00	0.00	0.01	0.01	0.00	0.00	0.00	0.00
Other	0.05	0.02	0.01	0.02	0.04	0.02	0.02	0.02	0.11	0.10	0.12	0.04	0.06	0.05	0.01
TOTAL READS	42943	48571	63114	55875	50945	64376	59271	56743	29812	79826	38852	39535	48406	46470	66834

Table includes taxa present with at least 1% abundance in two or more samples or with at least 5% abundance in one sample.
Taxa identified in the samples at low levels (< 1% abundance) are aggregated into category: "Other"
