# Supplementary material for: Comparison of the Vaginal Microbiomes of Premenopausal and Postmenopausal Women
Source: Front Microbiol. 2019 Feb 14;10:193. doi: 10.3389/fmicb.2019.00193 (PMC6382698; doi:10.3389/fmicb.2019.00193)
Supplement: Supplementary file 4 [file Table_4.PDF]

Table S4. Relative abundance of bacterial taxa based on 16S *rRNA* gene sequencing in vaginal samples obtained from postmenopausal women (POST).

	Subject														
**Taxon name**	**1001**	**1002**	**1004**	**1006**	**1007**	**1008**	**1009**	**1010**	**1011**	**1012**	**1013**	**1019**	**1020**	**1023**	**1027**
Acidovorax	0.00	0.00	0.02	0.00	0.00	0.00	0.00	0.00	0.00	0.00	0.01	0.15	0.00	0.00	0.27
Actinobaculum	0.00	0.00	0.00	0.01	0.00	0.00	0.00	0.00	0.00	0.01	0.00	0.00	0.00	0.00	0.00
Actinomyces	0.00	0.02	0.00	0.03	0.00	0.00	0.00	0.01	0.00	0.00	0.02	0.00	0.00	0.00	0.00
Aerococcus	0.00	0.00	0.00	0.00	0.00	0.00	0.00	0.00	0.03	0.00	0.00	0.00	0.00	0.09	0.00
Alloscardovia	0.00	0.00	0.00	0.08	0.00	0.00	0.00	0.00	0.00	0.00	0.03	0.00	0.00	0.00	0.00
Anaerococcus	0.00	0.54	0.00	0.01	0.01	0.00	0.05	0.05	0.00	0.12	0.09	0.00	0.00	0.05	0.00
Aquabacterium	0.00	0.00	0.01	0.00	0.00	0.00	0.00	0.00	0.00	0.00	0.00	0.02	0.00	0.00	0.12
Atopobium	0.00	0.00	0.00	0.72	0.00	0.00	0.00	0.00	0.09	0.00	0.00	0.00	0.06	0.00	0.00
Bacteroides	0.00	0.00	0.00	0.00	0.02	0.00	0.00	0.00	0.00	0.02	0.00	0.00	0.00	0.00	0.00
Blautia	0.00	0.00	0.00	0.00	0.06	0.00	0.00	0.00	0.00	0.05	0.00	0.00	0.00	0.00	0.00
Campylobacter	0.00	0.13	0.00	0.00	0.00	0.00	0.01	0.00	0.00	0.00	0.00	0.00	0.00	0.00	0.00
Cloacibacterium	0.00	0.00	0.00	0.00	0.00	0.00	0.00	0.00	0.00	0.00	0.00	0.03	0.00	0.00	0.03
Clostridiales	0.00	0.00	0.00	0.00	0.04	0.00	0.00	0.00	0.00	0.02	0.00	0.00	0.00	0.00	0.00
Corynebacterium	0.00	0.04	0.08	0.01	0.03	0.00	0.00	0.05	0.00	0.01	0.27	0.00	0.00	0.00	0.06
Dialister	0.00	0.00	0.00	0.01	0.00	0.00	0.08	0.00	0.00	0.01	0.00	0.00	0.00	0.00	0.00
Diaphorobacter	0.00	0.00	0.00	0.00	0.00	0.00	0.00	0.00	0.00	0.00	0.00	0.03	0.00	0.00	0.05
Facklamia	0.00	0.00	0.00	0.00	0.00	0.00	0.00	0.00	0.00	0.01	0.02	0.00	0.00	0.00	0.00
Faecalibacterium	0.00	0.00	0.00	0.00	0.14	0.00	0.00	0.00	0.00	0.04	0.00	0.00	0.00	0.00	0.00
Finegoldia	0.00	0.20	0.03	0.02	0.02	0.00	0.03	0.17	0.00	0.05	0.24	0.00	0.00	0.01	0.01
Gardnerella	0.93	0.00	0.11	0.00	0.03	0.00	0.00	0.00	0.35	0.00	0.00	0.02	0.70	0.00	0.02
Lachnospiracea_incertae_sedis	0.00	0.00	0.00	0.00	0.09	0.00	0.00	0.00	0.00	0.04	0.00	0.00	0.00	0.00	0.00
Lachnospiraceae	0.00	0.00	0.00	0.00	0.07	0.00	0.00	0.00	0.00	0.03	0.00	0.00	0.00	0.00	0.00
Lactobacillus	0.00	0.00	0.00	0.00	0.00	0.04	0.00	0.00	0.04	0.00	0.00	0.00	0.21	0.06	0.00
Lactobacillus crispatus	0.00	0.00	0.00	0.00	0.00	0.94	0.00	0.00	0.00	0.00	0.00	0.00	0.00	0.00	0.07
Lactobacillus iners	0.00	0.00	0.00	0.00	0.09	0.00	0.00	0.00	0.34	0.00	0.00	0.00	0.00	0.65	0.00
Lactobacillus jensenii	0.05	0.00	0.00	0.00	0.00	0.00	0.00	0.00	0.00	0.00	0.00	0.00	0.00	0.00	0.00
Oscillibacter	0.00	0.00	0.00	0.00	0.01	0.00	0.00	0.00	0.00	0.02	0.00	0.00	0.00	0.00	0.00
Peptoniphilus	0.00	0.00	0.02	0.01	0.01	0.00	0.07	0.05	0.00	0.08	0.04	0.00	0.00	0.03	0.00
Prevotella	0.00	0.00	0.00	0.02	0.00	0.00	0.54	0.03	0.00	0.05	0.02	0.00	0.00	0.04	0.01
Propionibacterium	0.00	0.00	0.01	0.00	0.06	0.00	0.00	0.00	0.00	0.00	0.00	0.00	0.00	0.00	0.00
Propionimicrobium	0.00	0.00	0.00	0.00	0.00	0.00	0.01	0.02	0.00	0.02	0.00	0.00	0.00	0.00	0.01
Ruminococcaceae	0.00	0.00	0.00	0.00	0.03	0.00	0.00	0.00	0.00	0.03	0.00	0.00	0.00	0.00	0.00
Ruminococcus	0.00	0.00	0.00	0.00	0.02	0.00	0.00	0.00	0.00	0.02	0.00	0.00	0.00	0.00	0.00
Staphylococcus	0.00	0.00	0.04	0.00	0.00	0.00	0.00	0.00	0.00	0.00	0.01	0.00	0.00	0.00	0.00
Streptococcus	0.00	0.00	0.21	0.03	0.01	0.00	0.06	0.51	0.00	0.04	0.09	0.48	0.00	0.00	0.04
Subdoligranulum	0.00	0.00	0.00	0.00	0.02	0.00	0.00	0.00	0.00	0.02	0.00	0.00	0.00	0.00	0.00
Varibaculum	0.00	0.00	0.00	0.00	0.00	0.00	0.02	0.00	0.00	0.06	0.01	0.00	0.00	0.00	0.00
Other	0.02	0.07	0.47	0.04	0.23	0.01	0.12	0.11	0.14	0.24	0.14	0.28	0.02	0.07	0.31
TOTAL READS	39894	31583	16540	45416	19891	40908	35478	27398	48105	38436	28163	12998	34601	44934	29880

Table includes taxa present with at least 1% abundance in two or more samples or with at least 5% abundance in one sample.
Taxa identified in the samples at low levels (< 1% abundance) are aggregated into category: "Other"
