# Supplementary material for: Comparison of the Vaginal Microbiomes of Premenopausal and Postmenopausal Women
Source: Front Microbiol. 2019 Feb 14;10:193. doi: 10.3389/fmicb.2019.00193 (PMC6382698; doi:10.3389/fmicb.2019.00193)
Supplement: Supplementary file 5 [file Table_5.PDF]

Table S5. Relative abundance of bacterial taxa based on 16S *rRNA* gene sequencing in vaginal samples obtained from postmenopausal women with hormone replacement therapy (POST+HT).

	Subject														
**Taxon name**	**1018**	**1040**	**1041**	**1042**	**1043**	**1044**	**1045**	**1046**	**1047**	**1051**	**1052**	**1054**	**1055**	**1056**	**1057**
Aerococcus	0.00	0.00	0.12	0.00	0.00	0.00	0.00	0.00	0.00	0.00	0.00	0.00	0.00	0.00	0.00
Atopobium	0.00	0.00	0.00	0.00	0.32	0.00	0.00	0.10	0.00	0.00	0.00	0.00	0.00	0.00	0.00
Bifidobacterium	0.00	0.00	0.73	0.00	0.00	0.00	0.00	0.00	0.00	0.00	0.00	0.00	0.00	0.00	0.78
Finegoldia	0.00	0.00	0.00	0.02	0.01	0.00	0.00	0.00	0.00	0.00	0.00	0.00	0.00	0.00	0.00
Gardnerella	1.00	0.64	0.00	0.00	0.00	0.00	0.00	0.85	0.00	0.00	0.00	0.00	0.00	0.00	0.00
Lactobacillus	0.00	0.02	0.02	0.04	0.03	0.04	0.04	0.00	0.06	0.04	0.04	0.04	0.09	0.04	0.00
Lactobacillus crispatus	0.00	0.00	0.00	0.85	0.00	0.95	0.91	0.00	0.73	0.94	0.95	0.93	0.00	0.94	0.00
Lactobacillus gasseri	0.00	0.00	0.00	0.00	0.50	0.00	0.02	0.00	0.00	0.00	0.00	0.00	0.00	0.00	0.00
Lactobacillus iners	0.00	0.32	0.00	0.00	0.00	0.00	0.00	0.00	0.16	0.00	0.00	0.00	0.71	0.00	0.00
Lactobacillus jensenii	0.00	0.00	0.00	0.00	0.00	0.00	0.00	0.00	0.00	0.00	0.00	0.00	0.18	0.00	0.00
Lactobacillus vaginalis	0.00	0.00	0.10	0.00	0.00	0.00	0.00	0.00	0.00	0.00	0.00	0.00	0.00	0.00	0.00
Prevotella	0.00	0.00	0.00	0.02	0.04	0.00	0.00	0.01	0.02	0.00	0.00	0.00	0.00	0.00	0.00
Staphylococcus	0.00	0.00	0.00	0.00	0.00	0.00	0.00	0.00	0.00	0.00	0.00	0.00	0.00	0.00	0.19
Other	0.00	0.03	0.03	0.07	0.09	0.01	0.03	0.04	0.03	0.02	0.01	0.02	0.02	0.01	0.03
TOTAL READS	73275	30320	54477	53657	42931	71710	41645	45863	39486	61873	71892	105331	71339	38923	40153

Table includes taxa present with at least 1% abundance in two or more samples or with at least 5% abundance in one sample.
Taxa identified in the samples at low levels (< 1% abundance) are aggregated into category: "Other"
