# Supplementary material for: Comparison of the Vaginal Microbiomes of Premenopausal and Postmenopausal Women
Source: Front Microbiol. 2019 Feb 14;10:193. doi: 10.3389/fmicb.2019.00193 (PMC6382698; doi:10.3389/fmicb.2019.00193)
Supplement: Supplementary file 6 [file Table_6.PDF]

Table S6. Vaginal atrophy scores, pH and bacterial community composition of postmenopausal women.

Vaginal atrophy [a]	Vaginal pH	Sample cluster [b]	Sample ID
7.0	6.0	B	1001
6.0	7.0	D	1002
9.0	7.0	D	1004
7.0	7.0	D	1006
6.0	7.5	D	1007
7.0	5.5	A	1008
8.0	8.0	D	1009
7.0	7.5	D	1010
7.0	6.5	B	1011
8.0	8.0	D	1012
8.0	8.0	D	1013
6.0	6.5	D	1019
7.0	6.0	B	1020
8.0	5.5	C	1023
7.0	7.5	D	1027
MEAN pH = 6.9 ± 0.7 [c]			
MEAN Atrophy score = 7.2 ± 0.7 [d]			

[a] 0-3 – no or little signs of vaginal atrophy, 4-5 – moderate vaginal atrophy, 6-8 – severe vaginal atrophy.

[b] Community groups A- D were characterized by having high proportions of *Lactobacillus crispatus*, *Gardnerella*, *Lactobacillus iners,* and anaerobic bacteria, respectively. Additional two smaller groups: E - *L. gasseri* or F - *Bifidobacterium*, contained only 2 samples in each group.

[c] Mean pH ± standard deviation

[d] Mean vaginal atrophy score ± standard deviation
