# Supplementary material for: Comparison of the Vaginal Microbiomes of Premenopausal and Postmenopausal Women
Source: Front Microbiol. 2019 Feb 14;10:193. doi: 10.3389/fmicb.2019.00193 (PMC6382698; doi:10.3389/fmicb.2019.00193)
Supplement: Supplementary file 7 [file Table_7.PDF]

Table S7. Vaginal atrophy scores, pH and bacterial community composition of postmenopausal women on HT.

Vaginal atrophy [b]	Vaginal pH	Sample cluster [c]	Sample ID
2.0	5.0	4	1018
0.0	4.5	4	1040
2.0	5.0	3	1041
2.0	4.0	1	1042
0.0	5.0	3	1043
1.0	4.0	1	1044
1.0	5.0	1	1045
0.0	4.5	4	1046
1.0	4.0	1	1047
1.0	5.0	1	1051
1.0	4.5	1	1052
0.0	4.0	1	1054
0.0	4.0	2	1055
0.0	4.0	1	1056
0.0	5.5	3	1057
MEAN pH = 4.5 ± 0.4 [c]			
MEAN Atrophy score = 0.7 ± 0.7 [d]			

[a] 0-3 – no or little signs of vaginal atrophy, 4-5 – moderate vaginal atrophy, 6-8 – severe vaginal atrophy.

[b] Community clusters 1- 4 were characterized by having high proportions of *Lactobacillus crispatus*, Lactobacillus *iners*, *Gardnerella,* and anaerobic bacteria, respectively.

[c] Mean pH ± standard deviation

[d] Mean vaginal atrophy score ± standard deviation
